# Imaging of benign salivary gland tumors: an update based on the fifth WHO classification, with a focus on rare histologic types and newly listed entities—part I

**DOI:** 10.1007/s11604-026-02063-w

**Published:** 2026-08-14

**Authors:** Tohru Takeshita, Taro Shimono, Hirotaka Takita, Daiju Ueda, Kayo Ueda, Yukio Miki, Akira Yamamoto

**Affiliations:** 1 Department of Radiology, Osaka Habikino Medical Center, 3-7-1, Habikino, Habikino City, Osaka 583-8588 Japan; 2 Department of Diagnostic and Interventional Radiology, Graduate School of Medicine, Osaka Metropolitan University, 1-4-3, Asahi-Machi, Abeno-ku, Osaka 545-8585 Japan; 3 Department of Artificial Intelligence, Graduate School of Medicine, Osaka Metropolitan University, Osaka, Japan

**Keywords:** Salivary gland tumors, WHO classification, Diagnostic imaging, Magnetic resonance imaging, Computed tomography

## Abstract

The salivary glands give rise to a diverse range of histologic tumor types. The World Health Organization (WHO) Classification of Head and Neck Tumors has been revised from the fourth edition (2017) to the fifth edition; the online version was released in March 2022, followed by the publication of the WHO Blue Book in March 2024. In the current classification of salivary gland tumors (SGTs), 15 benign and 21 malignant epithelial tumor types are categorized. Furthermore, four new benign entities were added: intercalated duct adenoma/hyperplasia, striated duct adenoma, sclerosing polycystic adenoma, and keratocystoma. Imaging features of prevalent benign tumors, such as pleomorphic adenoma, Warthin tumor, basal cell adenoma, salivary gland myoepithelioma, and oncocytoma, are thoroughly documented in the radiologic literature. Conversely, infrequently encountered and newly categorized benign tumors often remain unrecognized by many radiologists. In this Part I of a two-part series, we evaluate infrequently encountered benign SGTs within the current WHO classification, including cystadenoma of the salivary glands, canalicular adenoma, sialadenoma papilliferum, ductal papillomas, sebaceous adenoma, and lymphadenoma. The discussion focuses on diagnostic criteria with an emphasis on radiologic–pathologic correlations.

## Introduction

Salivary gland tumors (SGTs) exhibit marked histologic diversity, frequently complicating diagnosis on both histopathologic and radiologic assessment. Within the current World Health Organization (WHO) classification, 15 benign and 21 malignant epithelial tumor types are recognized (Table 1) [1–3]. The radiologic features of prevalent benign tumors, including pleomorphic adenoma (PA), Warthin tumor (WT), basal cell adenoma (BCA), salivary gland myoepithelioma (MYO), and oncocytoma (OC), have been thoroughly documented in the radiologic literature [4–31]. Conversely, rare benign tumors and newly categorized entities remain less familiar to general radiologists, and their radiologic characteristics are often documented only in limited reports.

**Table 1:** List of tumor types in the fifth edition of the World Health Organization Classification of Salivary Gland Tumors

Benign epithelial tumors	Malignant epithelial tumors
Pleomorphic adenoma	Mucoepidermoid carcinoma
Basal cell adenoma	Adenoid cystic carcinoma
Warthin tumor	Acinic cell carcinoma
Oncocytoma	Secretory carcinoma
Salivary gland myoepithelioma	Microsecretory adenocarcinoma^a^
Canalicular adenoma	Polymorphous adenocarcinoma
Cystadenoma of the salivary glands	Hyalinizing clear cell carcinoma
Ductal papillomas	Basal cell adenocarcinoma
Sialadenoma papilliferum	Intraductal carcinoma
Lymphadenoma	Salivary duct carcinoma
Sebaceous adenoma	Myoepithelial carcinoma
Intercalated duct adenoma and hyperplasia^a^	Epithelial-myoepithelial carcinoma
Striated duct adenoma^a^	Mucinous adenocarcinoma^a^
Sclerosing polycystic adenoma^a^	Sclerosing microcystic adenocarcinoma^a^
Keratocystoma^a^	Carcinoma ex pleomorphic adenoma
	Carcinosarcoma of the salivary glands
	Sebaceous adenocarcinoma
	Lymphoepithelial carcinoma
	Squamous cell carcinoma
	Sialoblastoma
	Salivary carcinoma, NOS and emerging entities

NOS, Not otherwise specified

^a^Newly listed entities in the salivary gland section of the fifth edition of the World Health Organization Classification of Head and Neck Tumors [1]

This article represents Part I of a two-part series regarding benign SGTs in the fifth WHO classification. Here, we evaluate six rare benign SGTs: cystadenoma (CA) of the salivary glands, canalicular adenoma (CAD), sialadenoma papilliferum (SP), ductal papillomas (DPs), sebaceous adenoma (SA), and lymphadenoma (LA), focusing on radiologic–pathologic correlation.

## Historical changes in the WHO classification of benign SGTs

**Fig. 1:**
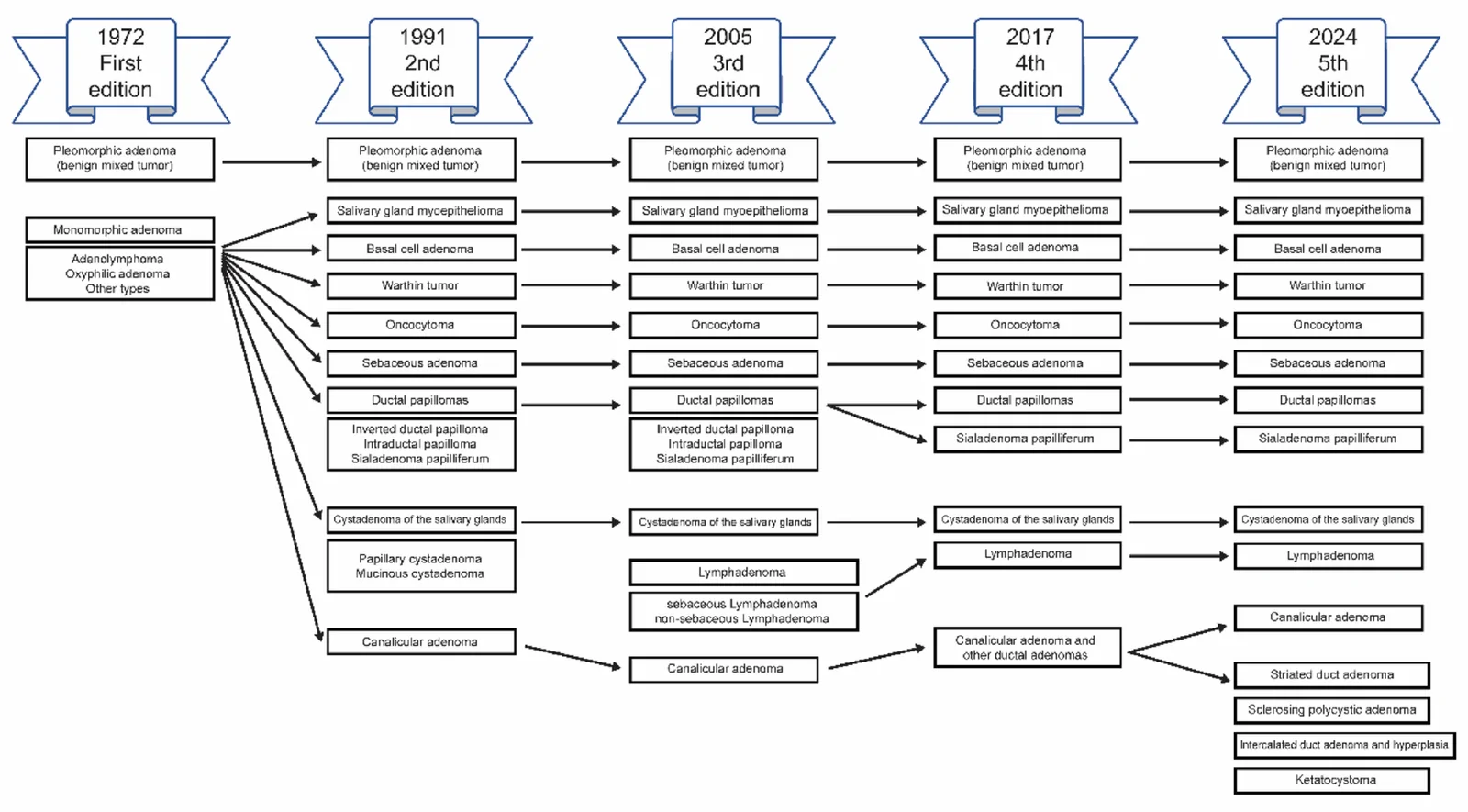
Historical changes in the World Health Organization (WHO) classification of benign epithelial salivary gland tumors. The diagram illustrates the evolution of entities from the first edition (1972) through the current fifth edition (2024) (adapted from [32])

The inaugural WHO classification (1972) categorized benign SGTs into two primary cohorts: PA and monomorphic adenoma [32]. The architectural subclassification of monomorphic adenoma included adenolymphoma (currently designated as WT), oxyphilic adenoma (currently designated as OC), and other tumors. The second edition (1991) expanded the benign SGT category to nine types and instituted a structural framework for the current histologic classification [32]. Subsequent minor refinements occurred in the third (2005) and fourth (2017) editions [32]. LA was introduced in the third edition, and SP was distinguished from DPs in the fourth edition [32]. Recently, advancements in molecular pathology, including novel genetic discoveries and immunohistochemical markers, combined with accumulated case-based data, culminated in the revisions of the fifth edition [1–3, 32] (Fig. 1).

## Benign SGTs affecting both major and minor salivary glands

In this section, CA and SA are discussed.

### Cystadenoma of the salivary glands

#### Definition

**Fig. 2:**
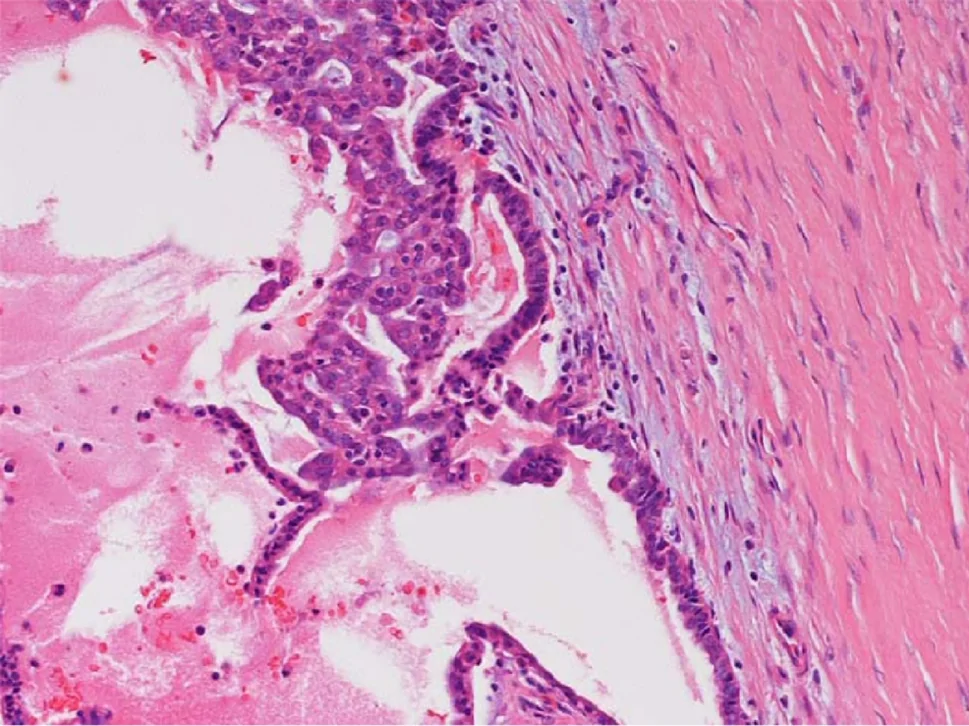
Histopathology of cystadenoma of the salivary glands. High-power photomicrograph (hematoxylin and eosin [H&E] stain) shows a multicystic architecture with a cuboidal epithelial lining and intraluminal papillary projections

CA is defined as a predominantly multicystic neoplasm characterized by a proliferative epithelial lining with variable papillary architecture and frequent oncocytic cytology (Fig. 2) [1].

#### Epidemiology and clinical presentation

CAs constitute 1%–4% of all SGTs [1]. CAs can involve both the major and minor salivary glands; the parotid gland is the single most common site, although collectively, the minor salivary glands may account for a comparable or greater proportion of cases [1, 33–37]. Meta-analytical data involving 126 cases [37] identified the parotid gland as the most frequent site (23.4%), followed by the palate (21.7%), buccal mucosa (15.6%), and upper lip (9.5%). Tumor dimensions range from 0.3 to 6.0 cm (mean: 1.5 cm) [37]. Patients demonstrate a broad age distribution, from infancy to 91 years (median age: 54 years), with a mild female predilection [37]. Clinically, CAs typically manifest as slow-growing, painless masses that may be difficult to distinguish from other cystic salivary lesions [1, 33–37]. Lesions within the minor salivary glands of the oral cavity may mimic mucoceles [1, 33–37].

#### Pathology

On macroscopic examination, CAs typically appear multilocular and contain cysts of varying dimensions [1, 33–37]. The cyst lining ranges from smooth and flat to granular or papillary, and cyst contents depend on the specific epithelial lining [1, 33–37]. On microscopic assessment, CAs are generally well-circumscribed, although they may occasionally entrap the adjacent salivary gland parenchyma [1, 33–37]. The cysts are separated by fibrous stroma and lined with various combinations of columnar, cuboidal, oncocytic, or flattened epithelial cells [1, 33–37]. Squamous, mucinous, apocrine, and ciliated cells are uncommon and, when present, typically constitute only a minor component [1, 33–37]. Three distinct histologic subtypes are categorized: papillary, oncocytic, and mucinous [1, 33–37]. The papillary subtype is the most prevalent [37] and is often concurrent with the oncocytic subtype [1, 33–37]. Oncocytic CAs resemble WT but lack lymphoid stroma; they demonstrate simple intraluminal papillary projections lined by one- or two-layered oncocytic cuboidal to columnar epithelium [1, 33–37]. Mucinous CAs are rare and exclusively composed of mucinous epithelial cells [1, 33–37]. Cytologic atypia, increased mitotic activity, complex papillary architecture, and invasive growth are uniformly absent [1, 33–37].

Immunohistochemically, the lining cells exhibit a luminal epithelial phenotype, with CK8/18 positivity and a p63-positive basal cell layer [1, 33–37]. S100 and SOX10 are usually negative or only focally positive [1, 33–37]. Oncocytic CAs exhibit BSND positivity; BSND is a novel immunohistochemical marker for striated duct cells and oncocytic tumor cells [38]. No defining molecular alterations have been established for CA subtypes [1, 39].

The histologic differential diagnosis encompasses other cystic SGTs, particularly cystic malignant neoplasms such as mucoepidermoid carcinoma (MEC), intraductal carcinoma, acinic cell carcinoma, secretory carcinoma, and mucinous adenocarcinoma (MA) [1, 36]. Table 2 summarizes the clinicopathologic features of CAs.

**Table 2:** Summary of the clinicopathologic features of cystadenoma of the salivary glands, sebaceous adenoma, and lymphadenoma

Feature	Cystadenoma of the salivary glands	Sebaceous adenoma	Lymphadenoma
Age and sex	Infancy–91 years (median: 54 years) Slight female predominance	22–90 years (peak incidence in the 6th decade) M:F ratio 4:3	10–78 years (median: 59 years) M:F ratio 4:3 Non-sebaceous type: slight female predominance
Location	Major ≈ minor salivary glands Parotid gland (most common)	Major > minor salivary glands Parotid gland (most common)	Parotid gland (majority)
Clinical appearance	Parotid mass or submucosal nodule (if of minor salivary gland origin)	Parotid mass	Parotid mass
Tumor border and encapsulation	Well-circumscribed Fibrous capsule (usually present)	Well-circumscribed Fibrous capsule (present)	Well-circumscribed Fibrous capsule (present)
Macroscopic features and size	Unicystic or multicystic 3–60 mm (mean: 15 mm)	Solid or solid-and-microcystic 4–60 mm	Solid, solid-and-microcystic, or multicystic Non-sebaceous: 6–50 mm (mean: 15 mm) Sebaceous: 10–60 mm (mean: 29 mm)
Histopathology and growth pattern	Mostly multicystic; intraluminal papillary projections	Sebaceous cell nests within a fibrous stroma	Admixture of epithelial and lymphoid components
Cytomorphology	Variable (bland); cuboidal, columnar, oncocytic, or mucinous	Sebaceous (bland)	Variable (bland); basaloid, squamoid, lymphoid, and sebaceous (sebaceous subtype only)
Stroma	Scant	Fibrous	Lymphoid (abundant)
Characteristic IHC staining	BSND (+) if oncocytic cells predominate	EMA, androgen receptor, adipophilin	No known defining markers
Tumor-specific gene alterations	No known defining mutations	No known defining mutations	No known defining mutations

BSND, Barttin CLCNK type accessory subunit beta; EMA, epithelial membrane antigen; IHC, immunohistochemical; M:F, male-to-female

BSND is a novel marker of the striated duct cells of normal salivary glands and oncocytic tumor cells. Nodular oncocytosis, oncocytoma, oncocytic cystadenoma, and Warthin tumor are typically positive for this marker [38]

#### Imaging findings

Comprehensive radiologic cohorts for CA are currently lacking. Takita et al. [40] described magnetic resonance imaging (MRI) findings of a left parotid CA as a well-demarcated cystic lesion with internal septations. Small mural projections extended into the cyst lumen, and the cyst contents exhibited hyperintensity on both T1-weighted imaging (T1WI) and T2-weighted imaging (T2WI).

### Sebaceous adenoma

#### Definition

**Fig. 3:**
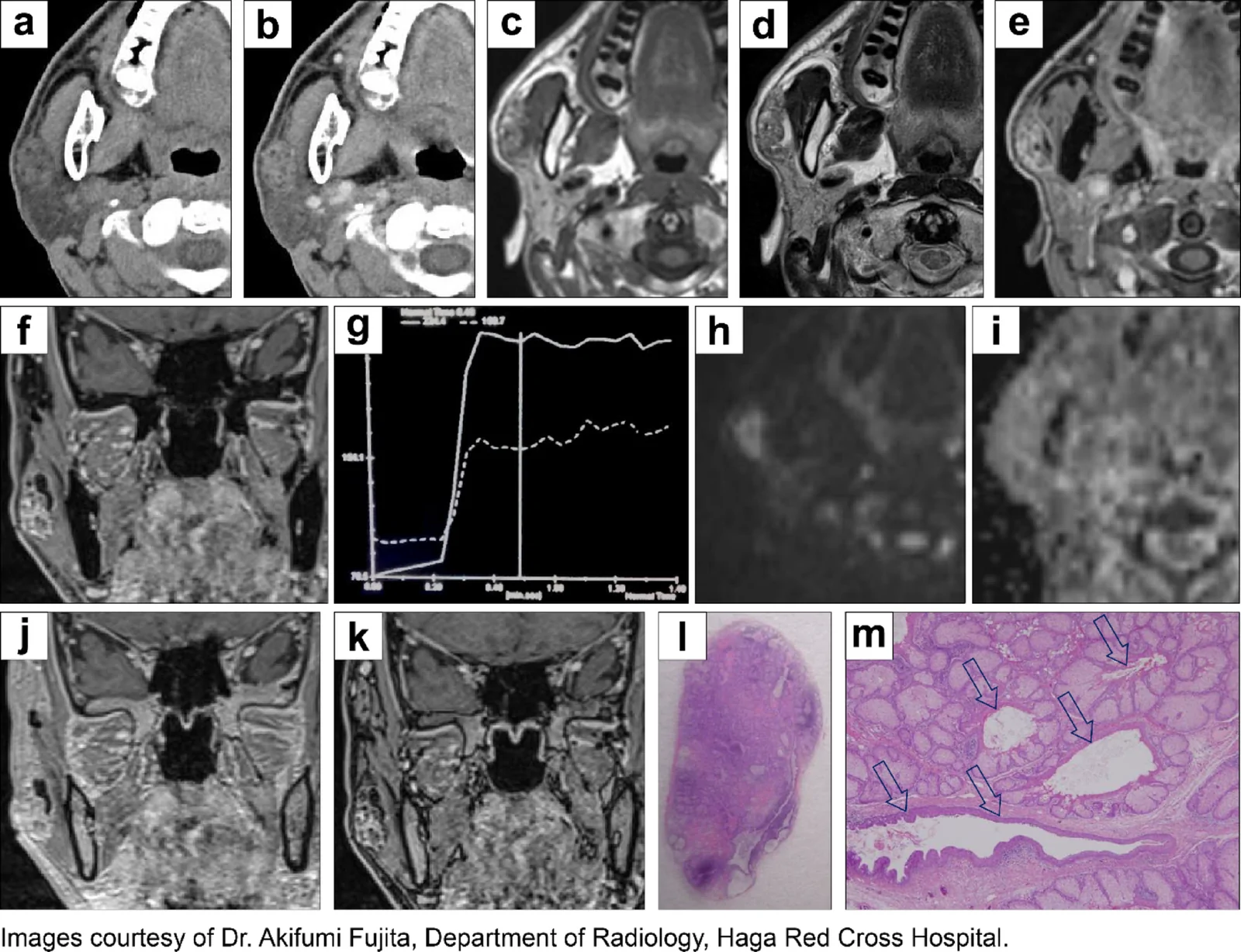
Imaging and histopathology of sebaceous adenoma in a man in his 40s. **a** Non-contrast-enhanced computed tomography (CT) demonstrates a soft-tissue-density mass with well-defined, smooth margins in the right parotid gland. Small, island-like low-density foci within the lesion correspond to fat. **b** Contrast-enhanced CT shows heterogeneous enhancement. **c** T1-weighted magnetic resonance (MR) imaging shows heterogeneous low-to-high signal intensity with small internal hyperintense foci. **d** T2-weighted MR imaging shows heterogeneous low-to-high signal intensity with small cyst-like and tubular hyperintense structures. **e**, **f** Axial (**e**) and coronal (**f**) gadolinium-enhanced T1-weighted MR imaging demonstrates heterogeneous enhancement. **g** Time–signal intensity curve on dynamic contrast-enhanced (DCE) MR imaging shows a rapid enhancement followed by a plateau pattern. **h**, **i** Diffusion-weighted imaging (DWI, h) and the corresponding apparent diffusion coefficient (ADC) map (**i**) show no evidence of significant diffusion restriction. **(j**, **k)** Coronal in-phase (**j**) and opposed-phase (**k**) chemical-shift MR images. The opposed-phase image demonstrates focal signal loss within the lesion (**k**), confirming the presence of microscopic fat. **l** Loupe view (hematoxylin and eosin [H&E] stain) shows a well-circumscribed tumor surrounded by a thin fibrous capsule. **m** High-power photomicrograph (H&E stain) shows a proliferation of epithelial cells with sebaceous differentiation forming nests of variable size and shape. The sebaceous cells exhibit characteristic clear, vacuolated cytoplasm. Small cystic spaces and duct-like structures are present within and around the tumor nests (arrows).

SA is defined as a well-demarcated tumor composed of irregular nests of sebaceous cells without cytologic atypia, frequently accompanied by cystic changes and squamous differentiation (Fig. 3) [1].

#### Epidemiology and clinical presentation

SA is rare, representing approximately 0.1% of all SGTs and slightly < 0.5% of all salivary gland adenomas [1]. Approximately 60% of cases arise within the major salivary glands, typically the parotid gland, whereas 40% occur in the minor salivary glands, particularly the buccal mucosa [1]. The mean age at presentation is within the sixth decade, with a reported range of 22–90 years and a male-to-female ratio of 4:3 [1]. Patients typically present with painless masses [1, 41–48]. Unlike cutaneous SAs, salivary gland SAs do not exhibit an association with Muir-Torre syndrome [41–48].

#### Pathology

On macroscopic examination, SAs are usually encapsulated or well-demarcated, exhibiting colors ranging from gray-white or pink–white to yellow–gray [1, 41–48]. These tumors range in diameter from 4 to 60 mm [1, 41–48]. On microscopic assessment, SAs are well-demarcated and encapsulated, comprising variably sized nests of monomorphic sebaceous cells, frequently featuring ductal or cystic changes [1, 41–48].

The ducts and cystic spaces may contain sebaceous material [1, 41–48]. Oncocytic metaplasia and squamous differentiation may also occur [1, 41, 42]. The stroma is typically fibrous, and histiocytes or foreign body giant cells attributable to extravasated sebum may be identified [1, 41–48]. Cellular atypia and pleomorphism are minimal, and invasion into adjacent tissues is unidentifiable [1, 41–48].

A characteristic cytologic feature of sebaceous neoplasms is cytoplasmic vacuolization caused by intracellular lipids [1, 49, 50]. Traditionally, special fat stains such as Oil Red O and Sudan IV on frozen sections have demonstrated lipid vacuoles, although immunohistochemistry is currently the preferred diagnostic method. Epithelial membrane antigen (EMA) and androgen receptor are robust markers of sebaceous differentiation [1, 49]. Adipophilin (ADP) facilitates the identification of intracellular lipid in formalin-fixed, paraffin-embedded tissue [1, 50]. No defining molecular alterations have been identified in SA [1].

The histologic differential diagnosis encompasses benign and malignant SGTs with sebaceous differentiation, including sebaceous carcinoma, epithelial-myoepithelial carcinoma with sebaceous differentiation, MEC with sebaceous differentiation, sebaceous LA, WT with sebaceous metaplasia, and PA with sebaceous metaplasia [1, 51]. Table 2 summarizes the clinicopathologic features of SAs.

#### Imaging findings

Radiologic data regarding SA are confined to case reports [43–45]. Shen et al. [43] described a right parotid SA as a mass exhibiting both fat and soft-tissue densities on computed tomography (CT), which histologically corresponded to sebaceous cell nests and fibrous stroma. Multiple fatty cysts with an attenuation value of approximately − 65 Hounsfield units (HUs) were identified; rupture of certain cysts resulted in the leakage of fatty material into the parapharyngeal space. Kim et al. [44] reported a right parotid SA manifesting as a well-demarcated spherical mass with low-attenuation, fat-containing components (approximately − 40 HUs) on noncontrast CT and thin peripheral capsular enhancement on contrast-enhanced CT. Welch et al. [45] described SA as a lesion with heterogeneous signal intensity and well-defined margins on T2WI.

In the current case of a right parotid SA (Fig. 3), the lesion was well-demarcated with smooth margins. Non-contrast-enhanced CT demonstrated a mass with soft-tissue density containing several small insular low-density foci corresponding to fat. Contrast-enhanced CT revealed heterogeneous enhancement. On both T1WI and T2WI, the lesion demonstrated heterogeneous low-to-high signal intensity. Small insular hyperintense foci were present on T1WI, and small cyst-like or tubular hyperintense structures were visualized on T2WI. Opposed-phase chemical-shift imaging revealed focal signal loss within the lesion. Significant diffusion restriction was unidentifiable on diffusion-weighted imaging (DWI) and apparent diffusion coefficient (ADC) maps. Dynamic contrast-enhanced (DCE) MRI exhibited a fast plateau pattern. On radiological-pathologic correlation, sebaceous tumor nests corresponded to the fat-containing components identified on CT and MRI, whereas the small cyst-like or tubular hyperintense structures on T2WI corresponded to the small cystic and duct-like structures within the tumor. The imaging differential diagnoses for SGTs containing fatty components include lipoma, sialolipoma, oncocytic lipoadenoma, PA or MYO with lipomatous metaplasia, sclerosing polycystic adenoma with fatty stroma, other tumors with sebaceous differentiation (e.g., sebaceous LA and sebaceous adenocarcinoma), and liposarcoma.

## Benign SGTs arising predominantly within the parotid or periparotid region

In this section, LA is discussed.

### Lymphadenoma

#### Definition

**Fig. 4:**
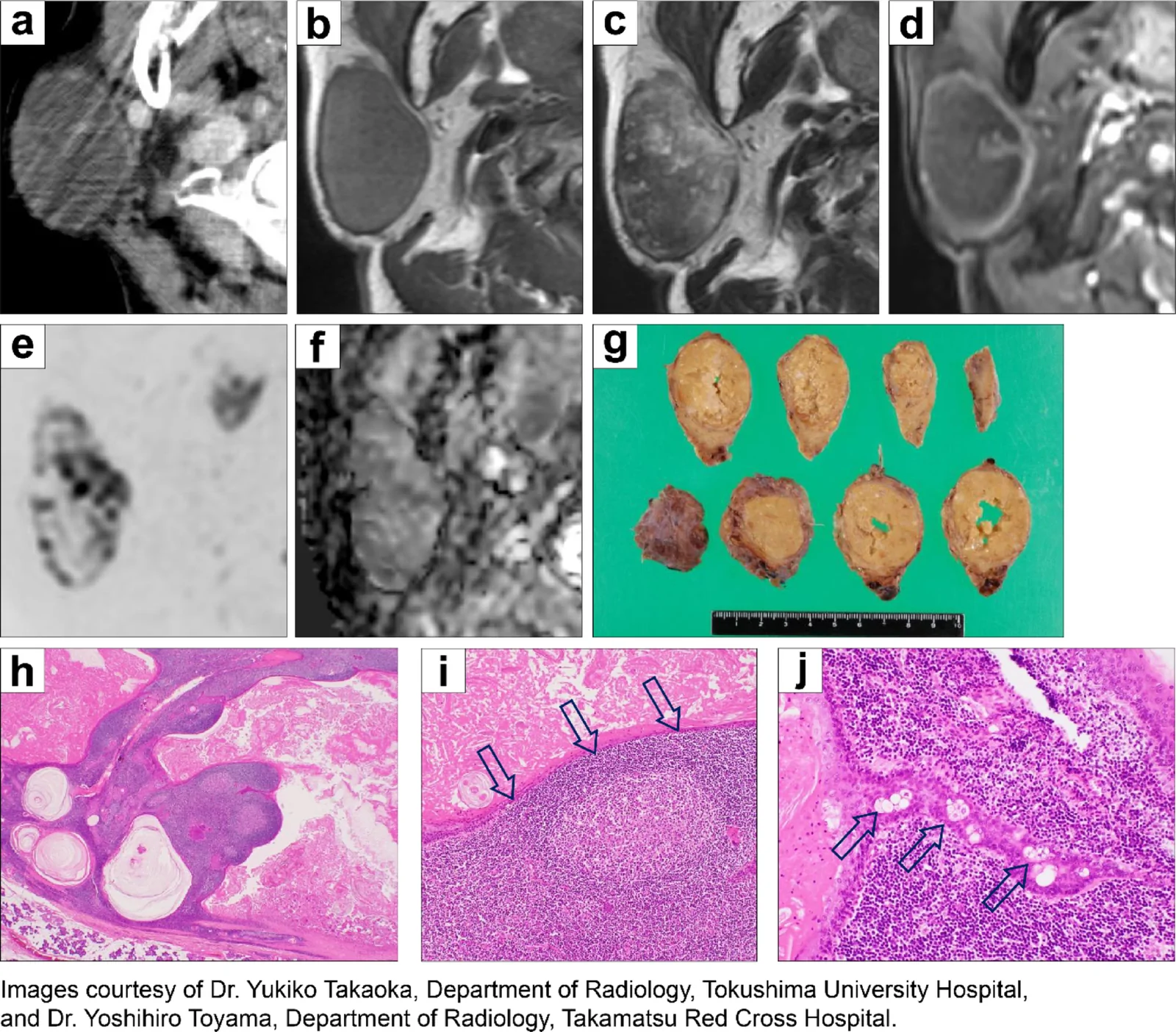
Imaging and histopathology of sebaceous lymphadenoma in a woman in her 60s. **a** Contrast-enhanced computed tomography (CT) demonstrates a mass with well-defined smooth margins in the right parotid gland. Thick capsular and septal enhancement is seen; however, the majority of the lesion shows poor enhancement. **b** T1-weighted magnetic resonance (MR) imaging shows low signal intensity with a prominent low-signal rim corresponding to the fibrous capsule. **c** T2-weighted MR imaging shows predominantly low signal intensity with focal hyperintense areas, resulting in a heterogeneous appearance. **d** Contrast-enhanced T1-weighted MR imaging confirms thick capsular and septal enhancement with poor central enhancement. **e**, **f** Axial diffusion-weighted whole-body imaging with background body signal suppression, displayed with inverted grayscale contrast (**e**), and the corresponding apparent diffusion coefficient (ADC) map (**f**) identify small, island-like abnormal foci with low ADC values. **g** Gross specimen (formalin-fixed). The cut surface of the parotid gland reveals a 50 × 50 × 34 mm yellowish mass. Cystic spaces contain sebaceous debris. **h** Low-power photomicrograph (hematoxylin and eosin [H&E] stain) shows a tumor with a clear border and a fibrous capsule. A dense lymphoid stroma with follicles is present, alongside cystic spaces filled with keratinous material and exudate. **i** High-power photomicrograph (H&E stain) highlights the reactive lymphoid stroma and germinal centers (arrows). **j** High-power photomicrograph (H&E stain) shows tumor cell trabeculae embedded within the lymphoid stroma. The epithelial component comprises squamoid and basaloid cells, with scattered cells showing sebaceous differentiation (arrows).

LA is defined as a benign SGT characterized by ductal or sebaceous epithelial nests situated within the reactive lymphoid stroma (Fig. 4) [1]. LAs are subclassified into sebaceous and non-sebaceous types, based on the presence or absence of sebaceous differentiation [1].

#### Epidemiology and clinical presentation

LAs are rare, accounting for approximately 0.1% of all SGTs and < 0.5% of salivary gland adenomas [1]. The sebaceous type is encountered more frequently than the non-sebaceous type [1]. Non-sebaceous LAs typically involve the parotid gland or adjacent periparotid tissue [1, 52–59]. Similarly, sebaceous LAs predominantly arise in the parotid or periparotid region, although rare occurrences have been documented in the submandibular gland, lacrimal gland, and minor salivary glands [1, 60–65]. Reported patient ages range from 10 to 78 years (median age: 59 years) [1, 52–65]. Non-sebaceous LAs tend to affect younger patients, including children [1]. Sebaceous LAs have a male-to-female ratio of approximately 4:3, whereas non-sebaceous LAs exhibit a slight female predilection [1]. Patients typically present with a slow-growing, painless mass [1, 52–65].

#### Pathology

On macroscopic examination, LAs are typically well-demarcated and encapsulated [1, 52–65]. They appear gray-white, tan, or yellow and may be solid or multicystic [1, 52–65]. Cysts frequently contain gelatinous or sebaceous materials [1, 52–65]. Sebaceous LAs are generally larger than non-sebaceous LAs, with mean dimensions of 29 mm (range: 10–60 mm) and 15 mm (range: 6–50 mm), respectively [1].

On microscopic assessment, LAs consist of epithelial proliferation within dense lymphoid tissue, surrounded by a fibrous capsule [1, 52–65]. The epithelial component forms anastomosing cords and nests of basaloid cells, concurrent with glandular, tubular, or cystic structures [1, 52–65]. Cysts vary in size and may contain eosinophilic hyaline material resembling the basement membrane [1, 52–65]. The degree of glandular and cystic formation varies between cases [1, 52–65]. Squamous differentiation and oncocytic metaplasia may be identified [1, 52–65]. Cytologic atypia and pleomorphism are minimal, whereas necrosis and mitotic activity are infrequent [1, 52–65].

In sebaceous LAs, sebaceous differentiation within the epithelial component may be scarce or abundant [1, 60–65]. Sebum leakage may induce a foreign body reaction, resulting in lymphoplasmacytic infiltration within the stroma [1, 60–65]. In both types, the lymphoid component consists of reactive follicles with germinal centers [1, 52–65].

Immunohistochemically, basal cells exhibit CK5/6 and p63 positivity, whereas luminal cells express CK7, CK18, and CK19; sebocytes exhibit p63, EMA, and ADP positivity [1, 54–59, 63]. The lymphoid cells are polytypic, and the Ki-67 labeling index is typically low [1]. No defining molecular alterations have been identified for LA [1].

The histologic differential diagnosis includes lesions characterized by prominent lymphoid stroma, such as lymphoepithelial lymphadenitis, IgG4-related sialadenitis, lymphoepithelial cysts, WT, lymphoepithelial carcinoma, Warthin-like MEC, carcinoma showing thymus-like elements of the salivary gland, malignant lymphoma, and metastatic carcinoma within an intraparotid lymph node [1, 58, 66–68]. Table 2 summarizes the clinicopathologic features of LAs.

#### Imaging findings

Radiologic reports regarding LA remain limited. Honda et al. described a sebaceous LA in the right parotid gland as a mass containing both fat and soft-tissue densities or signal intensities, reflecting variable proportions of sebaceous cell nests and lymphoid stroma [61]. A thick capsule was identified as rim enhancement on contrast-enhanced CT and MRI, and DCE-MRI demonstrated early enhancement with rapid washout. Yang et al. [65] reported multiple sebaceous LAs in the left parotid gland; the largest lesion exhibited heterogeneous hyperintensity on DWI, corresponding to a mixture of epithelial nests or trabeculae and dense lymphoid stroma.

In the current case of a sebaceous LA in the right parotid gland (Fig. 4), contrast-enhanced CT demonstrated thick capsular and septal enhancement, whereas the majority of the lesion exhibited poor enhancement. The lesion was hypointense on T1WI. On T2WI, most of the tumor was hypointense, with scattered hyperintense areas resulting in a heterogeneous appearance. A thick capsule was visualized on both T1WI and T2WI. Contrast-enhanced MRI demonstrated thick capsular and septal enhancement. On diffusion-weighted whole-body imaging with background body signal suppression, small insular foci of high signal intensity were visualized within the tumor and exhibited low ADC values. On radiologic–pathologic correlation, the fibrous capsule corresponded to the low-signal rim on both T1WI and T2WI and to the ring-like enhancement on contrast-enhanced CT and MRI. The cystic component containing proteinaceous material corresponded to areas of low signal intensity on both T1WI and T2WI. The tumor cell trabeculae embedded in abundant lymphoid stroma and sebaceous material corresponded to the abnormal foci identified on DWI.

## Benign SGTs arising predominantly within the intraoral minor salivary glands

In this section, CAD, SP, and DPs are discussed. CAD, SP, and DPs share an essential anatomic feature: they arise preferentially within the intraoral minor salivary glands [1, 69]. Many other SGTs predominantly involve the major salivary glands, particularly the parotid gland [1]. Consequently, identifying which tumor types preferentially arise within the intraoral minor salivary glands is clinically useful when estimating the histologic variant based on the site of origin [69]. Table 3 lists benign and malignant SGTs that occur predominantly in intraoral minor salivary glands and their common anatomic sites.

**Table 3:** Tumor types that occur predominantly in intraoral minor salivary glands and their common sites [69]

Tumor type	Common site(s)
Benign epithelial tumors	
Canalicular adenoma	Upper lip
Ductal papillomas	Lower lip, buccal mucosa, retromolar region
Sialadenoma papilliferum	Palate
Malignant epithelial tumors	
Hyalinizing clear cell carcinoma	Palate, tongue, lip, retromolar region
Mucinous adenocarcinoma	All minor salivary glands
Microsecretory adenocarcinoma	Palate, buccal mucosa
Polymorphous adenocarcinoma	Palate
Sclerosing microcystic adenocarcinoma	Tongue, palate, buccal mucosa

### Canalicular adenoma

#### Definition

**Fig. 5:**
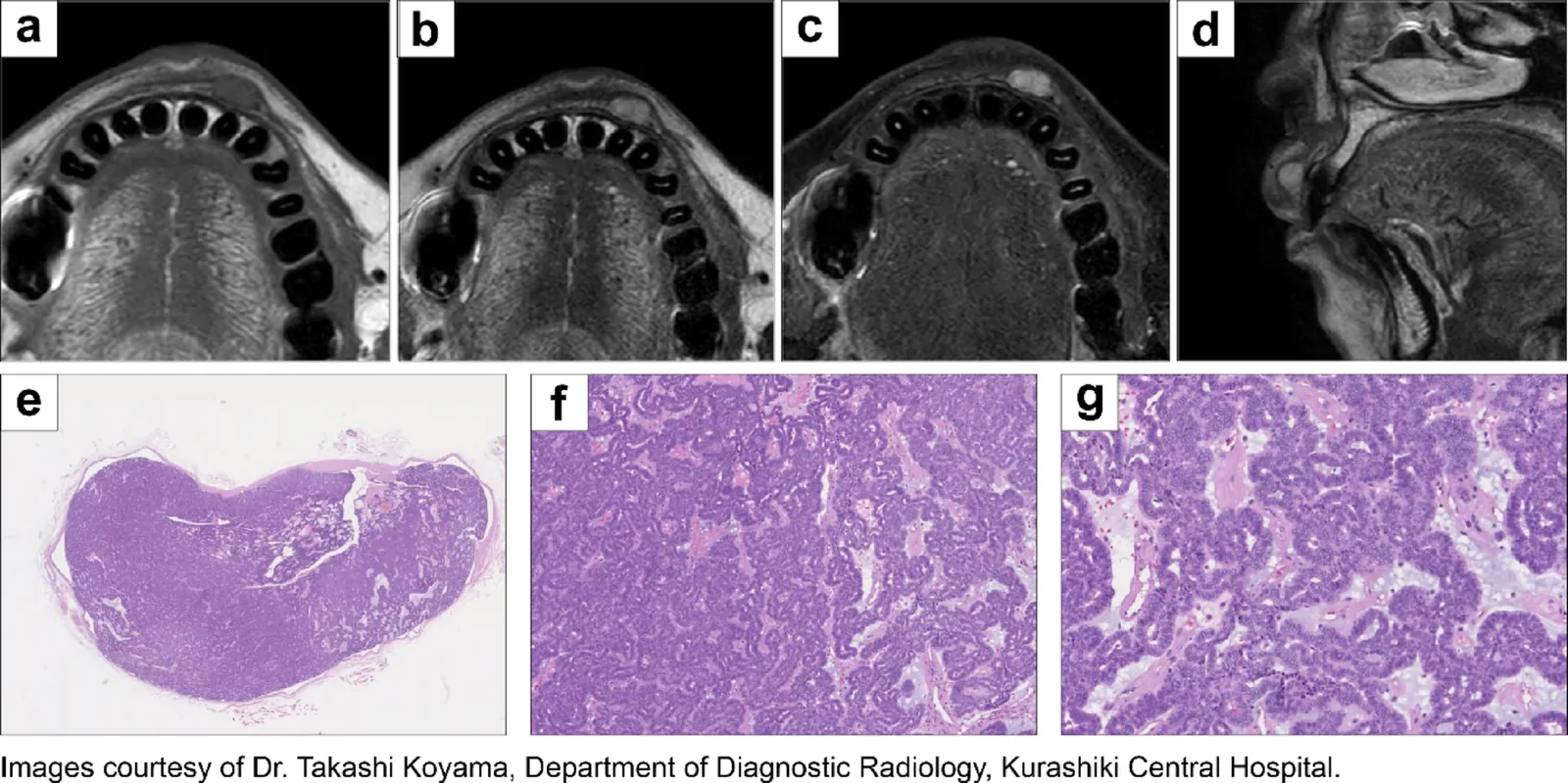
Imaging and histopathology of canalicular adenoma in a woman in her 70s. **a**–**d** Axial T1-weighted (a), axial T2-weighted (**b**), axial fat-suppressed T2-weighted (**c**), and sagittal T2-weighted (**d**) magnetic resonance (MR) imaging demonstrates a well-defined mass in the upper lip. The lesion is homogeneously hypointense on T1-weighted imaging and heterogeneously hyperintense on T2-weighted imaging. **e** Low-power photomicrograph (hematoxylin and eosin [H&E] stain) shows a well-circumscribed tumor with a pale, basophilic appearance. **f**, **g** Medium-power (**f**) and high-power (**g**) photomicrographs demonstrate a single layer of monotonous columnar epithelial cells arranged in a characteristic canalicular (“bead-on-a-string”) pattern. Basal and myoepithelial cell layers are absent. The supporting stroma is loose, edematous, and highly vascularized

CAD is defined as a benign salivary gland neoplasm composed of monomorphic epithelial cells arranged in anastomosing cords within a loosely vascularized stroma (Fig. 5) [1].

#### Epidemiology and clinical presentation

CAD was historically categorized as a subtype of BCA; however, it was established as an independent entity in the second edition of the WHO classification [32, 70, 71]. The nomenclature is derived from its characteristic histopathologic appearance, in which double rows of tumor cells create canal-like or “bead-on-a-string” structures [1, 71].

CAD represents < 1% of all SGTs and 4%–6% of benign minor salivary gland neoplasms [1]. Its reported frequency appears to vary according to the geographic region and population. In a study of 380 intraoral minor SGTs from California, CAD constituted 6.1% of benign tumors among 224 benign intraoral SGTs [72]. In contrast, a study of 737 intraoral minor SGTs from China and a study of 432 such tumors from Japan reported no CAD cases [73, 74]. CAD occurs almost exclusively within the intraoral minor salivary glands, particularly the upper lip, representing approximately 80% of cases, followed by the buccal mucosa, lower lip, and palate [1, 69, 75–80]. The reported age range is 33–91 years, with a peak incidence in the seventh decade and a slight female predilection [1, 69, 75, 80]. Patients typically present with a slow-growing, mobile, painless, non-ulcerated mass [1, 69, 75, 80]. Most cases are solitary, although synchronous multifocal lesions have been documented [77, 78]. In a clinicopathologic study of 67 cases, 9 (13.4%) were multifocal [75].

#### Pathology

On macroscopic examination, CADs are well-demarcated, solid, and frequently cystic; they appear as light yellow, tan, or brown translucent nodules [1, 69, 75–79]. They range in diameter from 2 to 30 mm and rarely exceed 30 mm [1, 69, 75–80]. On microscopic assessment, most lesions are lobulated or bosselated and are typically surrounded by a thin fibrous capsule [1, 69, 75–80]. The tumor cells comprise monotonous cuboidal to columnar cells with oval nuclei, evenly distributed chromatin, and frequent nuclear pseudostratification [1, 69, 75–80]. The cytoplasm is scant to moderate and eosinophilic, with indistinct cell borders [1, 69, 75–80]. The tumor cells form anastomosing single- or double-layered tubules and cords that repeatedly widen and narrow, resulting in a “bead-on-a-string” architectural pattern [1, 69, 75–80]. Intraluminal squamous balls or morules may be present within cystic spaces [1, 69, 75–80]. Basal and myoepithelial layers are uniformly absent [1, 69, 75–80]. The stroma is typically loose, hypocellular, and variably edematous to myxoid, despite its rich vascularity [1, 69, 75–80]. Laminated microliths are frequently present within the stroma [1, 69, 75–80].

Immunohistochemically, the tumor cells exhibit pan-cytokeratin, CK7, S100, SOX10, and GFAP positivity [1, 69, 75–80]. Luminal squamous balls may be highlighted by CK5/6 or p16 [1, 75–80]. Myoepithelial markers, such as smooth muscle actin, p63, calponin, and high-molecular-weight cytokeratin, are negative [1, 69, 75–80]. The stroma may exhibit CD117 positivity (c-kit), and the Ki-67 labeling index is typically low [1, 69, 75–80]. No defining molecular alterations have been established for CAD [1, 81].

The primary histologic differential diagnosis is striated duct adenoma, as both are unilayered ductal tumors composed of a single cell population [1–3, 80, 81]. Other differential diagnoses include PA, trabecular MYO, BCA, intercalated duct adenoma, polymorphous adenocarcinoma, and adenoid cystic carcinoma [1–3, 69, 75, 80, 81]. Table 4 summarizes the clinicopathologic features of CAD.

**Table 4:** Summary of the clinicopathologic features of canalicular adenoma, sialadenoma papilliferum, and ductal papillomas

Feature	Canalicular adenoma	Sialadenoma papilliferum	Ductal papillomas (intraductal)	Ductal papillomas (Inverted)
Location	Minor salivary glands; upper lip (most common)	Minor salivary glands; palate (most common)	Minor salivary glands; lower lip, buccal mucosa, retromolar region	Minor salivary glands; lower lip, buccal mucosa, retromolar region
Age and sex	33–91 years (peak: 7th decade) M:F ratio 1:1.7 Slight female predominance	2–91 years (median: 62 years) Peak: 5th–7th decades M:F ratio 1.5:1 Slight male predominance	Primarily adults No sex predilection	Primarily adults No sex predilection
Clinical appearance	Submucosal nodule in the upper lip	Exophytic papillary lesion of the palate	Submucosal nodule	Submucosal nodule
Tumor border and encapsulation	Well-circumscribed Fibrous capsule (present)	Unencapsulated	Well-circumscribed	Well-circumscribed Unencapsulated
Macroscopic features and size	Solid (partially cystic) 2–30 mm	Exophytic papillary < 10 mm (usually)	Unicystic < 15 mm	Solid (partially cystic) < 15 mm
Histopathology and growth pattern	Double columns of cells forming interconnecting strands and tubules in abundant stroma; “beaded appearance”	Combination of two components: surface exophytic papillary and submucosal ductal (multicystic with papillary proliferation)	Unicystic; intraluminal papillary proliferation	Inverted growth into submucosal tissue; broad, bulbous papillary proliferation; contiguous with surface mucosa
Cytomorphology	Monotonous high cuboidal to columnar cells; scant to moderate eosinophilic cytoplasm; oval, deep basophilic nuclei	Surface: squamous cells Submucosal: inner cuboidal/columnar cells and outer basal cells	Cuboidal or columnar cells with occasional mucous cells	Mixture of squamous, basaloid, cuboidal, columnar, and mucous cells
Stroma	Abundant; loose, paucicellular, edematous, and/or myxoid; highly vascularized	Fibrovascular core in papillary component; inflammatory cell infiltrate in submucosal stroma	Fibrovascular core within papillae	Fibrovascular core within papillae
Characteristic IHC staining	S100 (+, diffuse) Specific myoepithelial markers (−)	BRAF V600E (+) in both squamous and ductal cells; SOX10 (+) in ductal cells; p63 (+) in basal cells	No known defining markers	No known defining markers
Molecular genetics	No known defining mutations	*BRAF* V600E *HRAS* Q61R	No known defining mutations	*HRAS* G13R (rare; one case report)

IHC, immunohistochemical; M:F, male-to-female

#### Imaging findings

Detailed radiologic data for CAD remain limited. Yamada et al. [82] reported a case within the buccal mucosa that manifested as a well-demarcated, sharply marginated lesion exhibiting intense enhancement on contrast-enhanced CT, low signal intensity on T1WI, and high signal intensity on T2WI.

In the current case of CAD within the upper lip (Fig. 5), the lesion exhibited a clear margin and a mildly lobulated contour. It demonstrated low signal intensity on T1WI and heterogeneous high signal intensity on T2WI. On radiologic–pathologic correlation, the abundant edematous stroma likely contributed to the high signal intensity on T2WI.

### Sialadenoma papilliferum

#### Definition

**Fig. 6:**
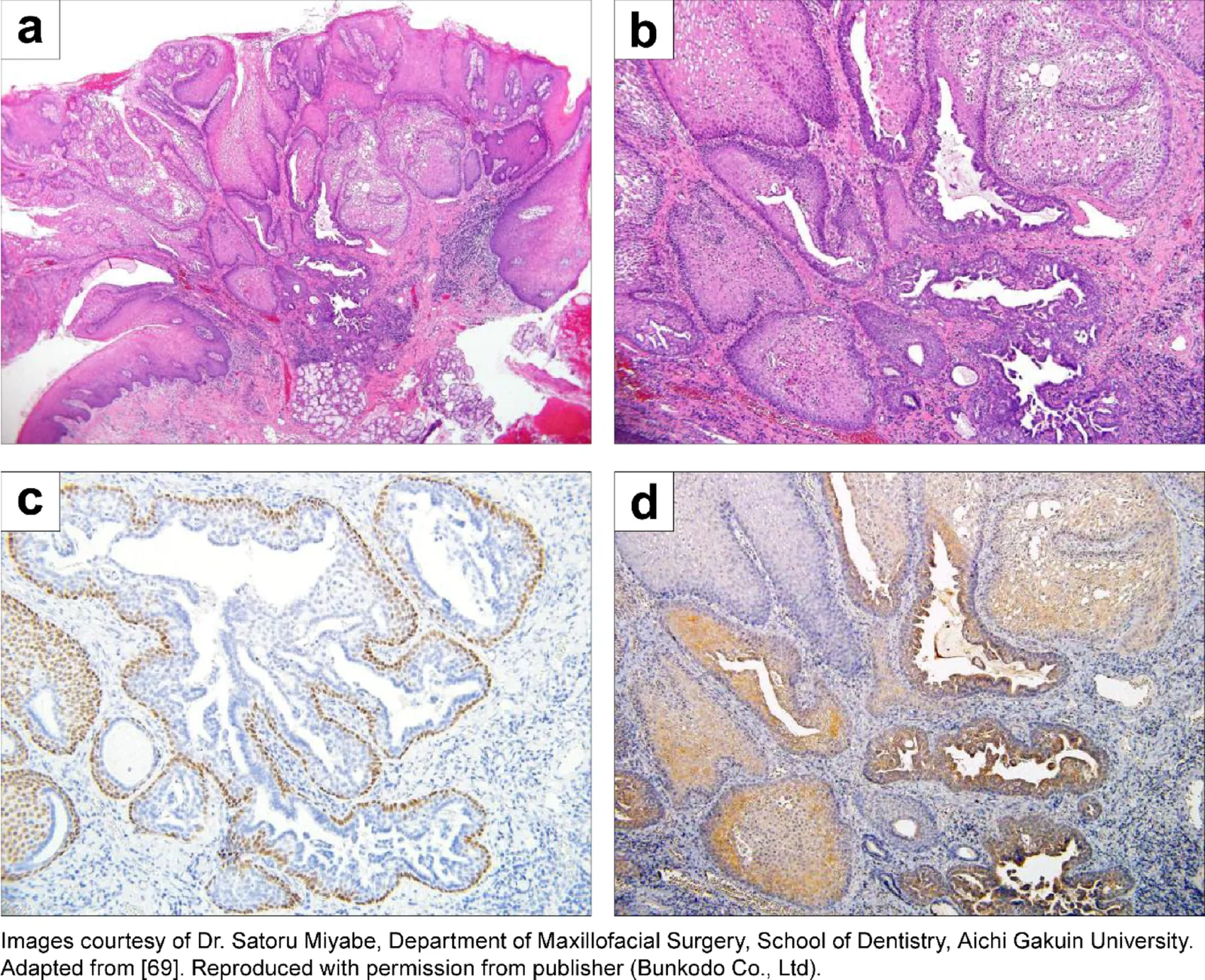
Histopathology of sialadenoma papilliferum. **a** Low-power photomicrograph (hematoxylin and eosin [H&E] stain) demonstrates a biphasic architecture: superficial exophytic squamous epithelial proliferation and underlying endophytic ductal proliferation. **b** High-power photomicrograph (H&E stain) of the ductal component shows cystically dilated ducts with irregular papillary epithelial proliferation. **c** Immunohistochemical (IHC) staining for p63 highlights a continuous layer of basal cells around the ducts, confirming a bilayered ductal epithelium. **d** BRAF V600E mutation-specific IHC staining shows positive expression in both the squamous and ductal components

SP is defined as a polypoid neoplasm characterized by both exophytic and endophytic (inward) papillary proliferation of the mucosal and salivary duct epithelium (Fig. 6) [1].

#### Epidemiology and clinical presentation

SP was initially described by Abrams and Finck in 1969 and was designated based on its morphological similarity to the syringocystadenoma papilliferum of the skin [83]. Historically, SP, inverted DP (IDP), and intraductal papilloma (IP) were collectively categorized as DPs in the second edition of the WHO classification [32, 70, 71]. In the fourth and fifth editions, SP was recognized as a distinct entity because of its unique histologic, immunohistochemical, and molecular features [32]. DPs are currently subdivided into IDP and IP [1, 32].

SP is rare; according to data from the Armed Forces Institute of Pathology, SP constitutes 0.4% of all minor SGTs [84]. It occurs most frequently in the hard palate, followed by the buccal mucosa [84–88]. Other documented sites include the soft palate, tongue, floor of the mouth, parotid gland, nasopharynx, esophagus, and bronchus [89–91]. Patients range in age from 2 to 91 years, with a median age of 62 years and a peak incidence between the fifth and seventh decades [1, 86]. A slight male predilection exists, with a male-to-female ratio of approximately 1.5:1 [1, 86]. Clinically, SP presents as a raised sessile or pedunculated mucosal lesion and may be mistaken for squamous papilloma, verrucous carcinoma, or exophytic DPs [1, 84–88].

#### Pathology

On macroscopic examination, SP is a well-demarcated papillary or pebbly lesion with a sessile or pedunculated base [1, 39, 69, 84–88]. Both the exophytic papillary component and the endophytic nodular base typically measure < 10 mm [1, 39, 69, 84–88]. Small cystic spaces are occasionally identifiable [1, 39, 69, 84–88]. Histologically, SP exhibits a characteristic biphasic architecture combining exophytic papillary squamous proliferation on the surface with contiguous endophytic salivary ductal epithelial proliferation beneath the mucosa [1, 39, 69, 84–88]. The exophytic surface is covered by parakeratinized squamous epithelium, and elongated branching ducts merge with the surface epithelium, forming clefts between papillary projections [1, 39, 69, 84–88]. Inward ductal proliferation is unencapsulated and typically consists of multiple small ducts with irregular luminal contours caused by epithelial folds or papillary projections, resulting in fissured or stellate lumina [1, 39, 69, 84–88]. The ducts are bilayered, featuring luminal cuboidal to columnar cells and abluminal cuboidal to flattened basal cells [1, 39, 69, 84–88]. Scattered mucous cells and focal oncocytic metaplasia may be identified [1, 39, 69, 84–88]. Variable numbers of inflammatory cells, particularly lymphocytes and plasma cells, are frequently observed within both the exophytic squamous and submucosal ductal components [1, 39, 69, 84–88].

Immunohistochemically, ductal cells exhibit CK7 and SOX10 positivity, whereas basal cells express p63. Exophytic squamous cells exhibit p63 and CK13 positivity and may demonstrate focal CK7 positivity [1, 39, 69, 84–88]. The *BRAF* p.V600E mutation is consistently reported in classic SP [1, 39, 69, 85–88]. Both the squamous and ductal components may demonstrate positive staining with a mutation-specific BRAF V600E antibody [1, 39, 69, 85–88]. Two subtypes have been proposed based on SOX10 expression and BRAF status: classic and oncocytic [85, 86]. Oncocytic SP exhibits the same architectural framework as classic SP, but its ductal component is entirely lined by oncocytic cells [85, 86]. In classic SP, the proliferating ductal cells are SOX10 positive and *BRAF* V600E mutated, whereas oncocytic SP is SOX10 negative and typically exhibits *BRAF* wild-type status [85, 86].

The primary histologic differential diagnoses include exophytic DP, IP, IDP, and papillary CA [1, 39, 84–88]. The coexistence of exophytic and endophytic growth is the key feature that differentiates SP from other papillary-cystic SGTs. Table 4 summarizes the clinicopathologic features of SP.

#### Imaging findings

As SP is rare and typically small in size, published radiologic reports remain sparse. Tominaga et al. [92] documented a case involving the hard palate in which both contrast-enhanced CT and MRI demonstrated a focally enhancing lesion.

### Ductal papillomas

#### Definition

**Fig. 7:**
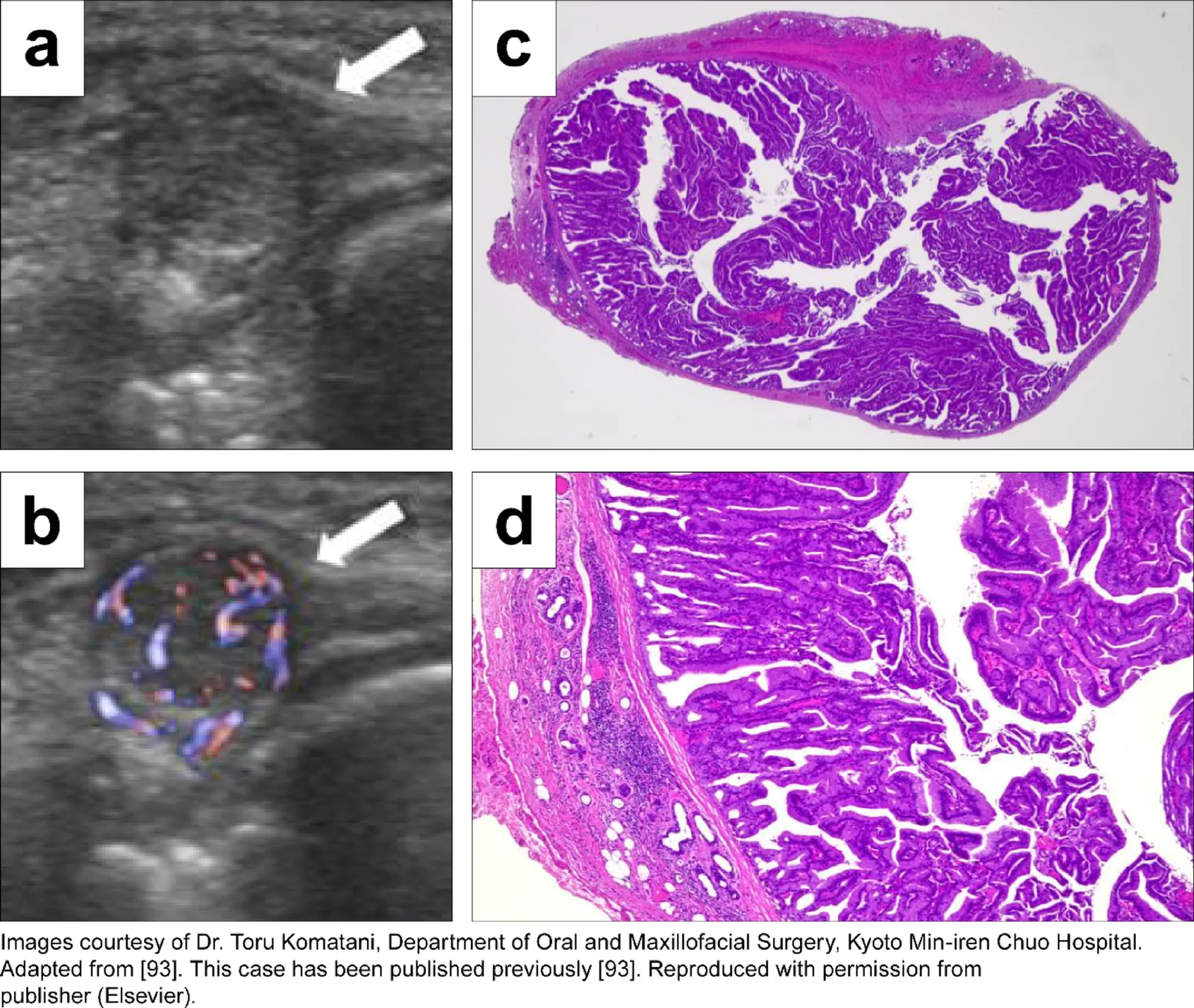
Imaging and histopathology of intraductal papilloma in a woman in her 40s. Adapted from [93]. **a** Ultrasonography demonstrates a cystic mass containing internal echogenic material (arrow). **b** Color Doppler ultrasonography shows internal vascular flow (arrow). **c** Low-power photomicrograph (hematoxylin and eosin [H&E] stain) shows a well-circumscribed, encapsulated, unilocular cystic mass filled with papillary epithelial proliferation. **d** High-power photomicrograph (H&E stain) reveals papillae with thin fibrovascular cores lined by bland, columnar epithelial cells

DPs are defined as benign epithelial neoplasms originating within the primary excretory salivary ducts (Fig. 7) [1].

#### Epidemiology and clinical presentation

DPs constitute approximately 0.3% of intraoral minor SGTs [1]. They originate most frequently within the minor salivary glands of the lower lip, buccal mucosa, and palate [1, 94]. Most patients are adults, with no significant sex predilection, although rare pediatric cases have been documented [1, 94]. Clinically, DPs manifest as slow-growing, painless submucosal nodular masses or swellings [1, 94].

#### Pathology

On macroscopic examination, most DPs are small, measuring < 15 mm [1]. On microscopic assessment, DPs are categorized into IP and IDP based on their respective growth patterns [1, 94]. IP originates at the salivary excretory duct and manifests as a unilocular cystic lesion featuring papillary projections [1, 39, 93, 95, 96]. The papillae are lined by columnar cells, with occasional mucinous cells also present [1, 39, 93, 95, 96]. Basal and myoepithelial layers are uniformly absent from the cyst wall and papillary fronds [1, 39, 93, 95, 96]. Cytologic atypia, mitotic activity, and invasive growth are unidentifiable [1, 39, 93, 95, 96]. IDP is a well-demarcated but unencapsulated tumor exhibiting endophytic, inverted growth and a globular configuration. IDP originates adjacent to the ostium of the salivary excretory duct, frequently associated with a punctum or dilated pore on the oral mucosal surface [1, 97–100]. It is composed of non-keratinizing squamous cells and mucous cells [1, 97–100]. Cytologic atypia is unidentifiable, and mitotic figures are infrequent [1, 97–100].

DPs are typically identified on routine standard hematoxylin and eosin-stained sections, and immunohistochemical analyses are infrequently necessary [1]. No defining molecular alterations have been identified for IP [1, 39]. A single case of IDP featuring an *HRAS* p.G13R mutation has been documented [98].

The histologic differential diagnosis encompasses other papillary tumors of the intraoral minor salivary glands, particularly SP and papillary CA [1, 39, 86, 98, 101]. Owing to the potential presence of mucous cells within DPs, MEC, and MA warrant clinical consideration [1, 39, 86, 98, 101]. Table 4 summarizes the clinicopathologic features of DPs.

#### Imaging findings

Radiologic descriptions of DPs remain sparse owing to the rarity and diminutive size of these lesions. Yamada [95] documented a case of IP involving the buccal mucosa that demonstrated heterogeneous high signal intensity on T2WI. Komatani et al. [93] reported an IP of the buccal mucosa that manifested on ultrasonography (US) as a markedly dilated duct with internal echogenicity. Color Doppler US demonstrated intralesional vascular flow (Fig. 7).

## Conclusion

In this review, we summarize six rare benign SGTs included in the current WHO classification and outline their clinicopathologic and multimodality imaging features. Representative images highlighting pivotal findings are presented, and Table 5 presents the salient radiologic features for the six tumor types evaluated. Familiarity with the current WHO classification and salivary gland pathology is essential; furthermore, radiologic–pathologic correlation is fundamental to achieving an accurate diagnosis.

**Table 5:** Key imaging findings of the six benign salivary gland tumors reviewed in this article

Tumor type	Key imaging findings
Cystadenoma of the salivary glands	• Well-circumscribed, multicystic mass with small papillary projections • Variable signal intensity of cyst contents on T1WI and T2WI
Sebaceous adenoma	• Well-circumscribed; may appear solid or solid-and-microcystic • Heterogeneous low-to-high signal intensity on T1WI and T2WI • Small areas of fat density (CT) or fat signal intensity (MRI) within the mass • Visible capsule on MRI • Capsular enhancement on contrast-enhanced CT and MRI
Lymphadenoma	• Well-circumscribed; may appear solid, solid-and-microcystic, or multicystic • Heterogeneous low-to-high signal intensity on T1WI and T2WI • Small areas of fat density/signal intensity within the mass (specifically in the sebaceous subtype or when sebaceous cells are abundant) • Heterogeneous high signal intensity on diffusion-weighted imaging • Visible capsule on MRI with capsular enhancement on contrast-enhanced CT and MRI • Early enhancement and rapid washout pattern on dynamic contrast-enhanced MRI
Canalicular adenoma	• Well-circumscribed, smooth or slightly lobulated mass, typically located in the upper lip • High signal intensity on T2WI • Intense enhancement on contrast-enhanced CT
Sialadenoma papilliferum	• Small enhancing mass on the palate, visualized on CT and MRI
Ductal papillomas (intraductal)	• Well-circumscribed, unicystic mass with heterogeneous high signal intensity on T2WI • Well-circumscribed, hypoechoic mass with internal echogenicity on ultrasonography • Internal vascular flow visible on color Doppler ultrasonography

CT, computed tomography; MRI, magnetic resonance imaging

## Data Availability

Data sharing is not applicable to this article as no datasets were generated or analyzed during the current study.

## Abbreviations

ADC: Apparent diffusion coefficient
ADP: Adipophilin
BCA: Basal cell adenoma
BRAF: B-raf proto-oncogene
BSND: Barttin CLCNK type accessory subunit beta
CA: Cystadenoma of the salivary glands
CAD: Canalicular adenoma
CK: Cytokeratin
CT: Computed tomography
DCE: Dynamic contrast-enhanced
DP: Ductal papilloma
DWI: Diffusion-weighted imaging
EMA: Epithelial membrane antigen
GFAP: Glial fibrillary acidic protein
H&E: Hematoxylin and eosin
HRAS: Harvey rat sarcoma viral oncogene homolog
HU: Hounsfield Units
IDP: Inverted ductal papilloma
Ig: Immunoglobulin
IP: Intraductal papilloma
LA: Lymphadenoma
MA: Mucinous adenocarcinoma
MEC: Mucoepidermoid carcinoma
MRI: Magnetic resonance imaging
MYO: Salivary gland myoepithelioma
OC: Oncocytoma
PA: Pleomorphic adenoma
SA: Sebaceous adenoma
SGT: Salivary gland tumor
SOX10: SRY-related HMG-box 10
SP: Sialadenoma papilliferum
US: Ultrasonography
WHO: World Health Organization
T1WI: T1-weighted imaging
T2WI: T2-weighted imaging
WT: Warthin tumor

## Declarations

### Generative AI in scientific writing

The authors used ChatGPT (GPT-5.4 Pro, OpenAI, San Francisco, CA, USA) for assistance with English proofreading.
